# Capsular profiling of the *Cronobacter* genus and the association of specific *Cronobacter sakazakii* and *C. malonaticus* capsule types with neonatal meningitis and necrotizing enterocolitis

**DOI:** 10.1186/s12864-015-1960-z

**Published:** 2015-10-08

**Authors:** P. Ogrodzki, S. Forsythe

**Affiliations:** Pathogen Research Group, School of Science and Technology, Nottingham Trent University, Clifton Lane, NG11 8NS, Nottingham, UK

**Keywords:** *Cronobacter*, Capsule formation, Genomic analysis

## Abstract

**Background:**

*Cronobacter sakazakii* and *C. malonaticus* can cause serious diseases especially in infants where they are associated with rare but fatal neonatal infections such as meningitis and necrotising enterocolitis.

**Methods:**

This study used 104 whole genome sequenced strains, covering all seven species in the genus, to analyse capsule associated clusters of genes involved in the biosynthesis of the O-antigen, colanic acid, bacterial cellulose, enterobacterial common antigen (ECA), and a previously uncharacterised K-antigen.

**Results:**

Phylogeny of the *gnd* and *galF* genes flanking the O-antigen region enabled the defining of 38 subgroups which are potential serotypes. Two variants of the colanic acid synthesis gene cluster (CA1 and CA2) were found which differed with the absence of *galE* in CA2. Cellulose (*bcs* genes) were present in all species, but were absent in *C. sakazakii* sequence type (ST) 13 and clonal complex (CC) 100 strains. The ECA locus was found in all strains. The K-antigen capsular polysaccharide Region 1 (*kpsEDCS*) and Region 3 (*kpsMT*) genes were found in all *Cronobacter* strains. The highly variable Region 2 genes were assigned to 2 homology groups (K1 and K2). *C. sakazakii* and *C. malonaticus* isolates with capsular type [K2:CA2:Cell^+^] were associated with neonatal meningitis and necrotizing enterocolitis. Other capsular types were less associated with clinical infections.

**Conclusion:**

This study proposes a new capsular typing scheme which identifies a possible important virulence trait associated with severe neonatal infections. The various capsular polysaccharide structures warrant further investigation as they could be relevant to macrophage survival, desiccation resistance, environmental survival, and biofilm formation in the hospital environment, including neonatal enteral feeding tubes.

**Electronic supplementary material:**

The online version of this article (doi:10.1186/s12864-015-1960-z) contains supplementary material, which is available to authorized users.

## Background

Capsular polysaccharides (CPS) are major bacterial virulence factors and environmental fitness traits. In Gram-negative bacteria, the CPS forms a surface layer of water-saturated, high molecular weight polysaccharides which enable the organism to evade host response mechanisms such as phagocytosis as well as facilitate biofilm formation and desiccation survival [1]. CPS vary considerably between organisms, and even between strains of the same species. Capsular diversity has been the basis for a number of bacterial differentiation methods including serotyping of *Salmonella* serovars and the K-antigen classification scheme of *E. coli* [2]. The use of whole genome data can now serve to expand and clarify these schemes. Such analysis can also reveal the extent of horizontal gene transfer leading to the lack of congruence between serotype and phylogeny due to the transference of loci (ie. *rfb* locus) between strains.

In Gram-negative bacteria the O-antigen and K-antigen are composed of long polysaccharide units which are covalently linked to lipid A in the outer membrane. The O-antigen is a major surface antigen. Genes involved in O-antigen synthesis are in the *rfb* locus between the flanking genes *gnd* and *galF* [3]. The locus varies in size for each serotype according to the sugar composition and complexity of structure, ie. phosphorylation. These genes encode for enzymes involved in the synthesis of sugars forming the O-antigen subunit, genes that encode glycosyltransferases (required for the assembly of sugar substituents in the O-antigen subunit) and genes such as *wzx* and *wzy*. The latter encode for the transporter and polymerase proteins necessary for processing and assembly of the O-antigen from the subunits.

The K-capsule of *E. coli* is composed of the highly conserved Regions 1 (*kpsFEDUCS*) and 3 (*kpsMT*), and a variable Region 2 [1, 2]. Regions 1 and 3 encode for enzymes and transport proteins responsible for initiation of chain elongation and translocation to the cell surface. Region 2 genes encode the glycosyltransferases and other enzymes responsible for biosynthesis of the K-antigen-specific CPS such as *neu* genes for the polysialic acid capsule in the neonatal meningitic *E. coli* K1 pathovar.

Many Gram-negative bacteria also secrete a variety of high molecular weight glycopolymers, known as exopolysaccharides (EPS), which contribute to biofilm formation [4, 5]. The enterobacterial common antigen (ECA) is a linear heteropolysaccharide which is bound to the outer membrane. It is composed of → 3)-α-DFuc*p*4NAc-(1 → 4)-β-D-Man*p*NAcA-(1 → 4)-α-DGlc*p*NAc-(1 → (2–4), modified with O-acetyl groups as a repeating trisaccharide unit. Three variations in ECA have previously been described; ECA_PG_ linked to phosphatidylglycerol, ECA_LPS_ covalently bound to the lipid A core structure, and ECA_CYC_ which is a periplasmic water-soluble cyclic form [6]. Most of the genes involved in the assembly of ECA are located in the *wec* gene cluster [7, 8]. Another exopolysaccharide, called colanic acid (CA) is loosely bound to the cell. CA may also be secreted into the environment where it contributes to bacterial biofilm structure [4]. *Enterobacteriaceae* also synthesize an exopolysaccharide known as bacterial cellulose, or poly-β-1,4-glucan, which forms part of the bacterial extracellular matrix [9]. Regulatory expression and secretion of these EPS can be influenced by various factors, for example the thermoregulated synthesis of polysialic acid and colanic acid in *E. coli* [10].

The bacterial pathogen *Cronobacter* has become the focus of much attention especially due to its association with neonatal meningitis [11]. A number of potential virulence traits have been proposed, most recently the production of outer membrane vesicles [12–14]. Most strains of *Cronobacter* are able to survive and even replicate inside macrophages for up to 2 days [15, 16]. The genus is composed of seven species, for which an open access international multilocus sequence typing database has been established; http://pubmlst.org/cronobacter/ [17–19]. This database has enabled the recognition of certain *Cronobacter* clonal lineages as pathovars; *C. sakazakii* clonal complex (CC) of ST4 are more predominantly associated with neonatal meningitis, *C. sakazakii* ST12 with neonatal necrotizing enterocolitis, and *C. malonaticus* ST7 with adult infections [13, 17–20]. However genome comparison studies have not revealed virulence traits unique to these *Cronobacter* pathovars [14, 20, 21].

In *Cronobacter* the O-antigen gene cluster contains between 6 and 19 genes, and varies between 6–20 kb in length. It is flanked by *gnd* and *galF* (encoding 6-phosphogluconate dehydrogenase and UTP-glucose-1-phosphate uridylyltransferase subunit, respectively) [21, 22]. The variation in the *rfb* cluster has been used to develop random fragment length polymorphism (RFLP) profiling across the region and PCR assays targeting the presumed conserved serotype-specific genes *wzx* and *wzy* [23, 24]. Until recently there were only 18 defined serotypes across the whole genus, with only 7 in *C. sakazakii* and 2 in *C. malonaticus*, respectively [22–27]. There are however contradictions in the literature such as *C. sakazakii* O5 and O6 being proposed as mis-identified strains of *C. malonaticus* [26, 27]. In addition, RFLP and PCR-probes are unable to detect all sequence based variations which may be outside their target site. Consequently, Blažková et al. [27], based on their more detailed analysis, proposed the O-antigen scheme should be expanded, with additional recognition of 7 new and 2 re-assigned serotypes. *Cronobacter* serotyping has been supported in part by chemical analysis of the O-antigen polysaccharide (O-PS) from many strains [28–32]. However three structures have been determined for *C. sakazakii* O2 strains [28–30]. This observation supports the proposal that the current RFLP and PCR-probes are unable to distinguish some variants in the O-antigen.

An additional complication with current O-antigen assignment is that the targeted genes do not follow whole genome phylogeny of the genus [14]. There is evidence that horizontal gene transfer has occurred in the *rfb* region. For example, the same O-antigen serotype sequences in the *wzx* and *wzy* genes are used as target sites for different *Cronobacter* species such as *C. malonaticus* O1 and *C. turicensis* O1 [24]. Also some homologies are found in different species and genera, as occurs with *C. sakazakii* O3 and *C. muytjensii* O1 with *E. coli* O29 and *C. sakazakii* O4 with *E. coli* O103 [24, 25, 32]. Therefore currently O-antigen serotyping can only be determined after the *Cronobacter* isolates have been first accurately speciated. However this too has been problematic due to the reliance on phenotyping as well as *rpoB* and *cgcA* PCR probes which have even mis-identified infant infections as *C. sakazakii* instead of the correct identification of *E. hormaechei* [33, 34].

Given the smaller number of serotypes than MLST defined sequence types (24 compared to >350) it is not unexpected that several sequence types occur in each serotype. Based on >1000 entries in the *Cronobacter* PubMLST database, *C. sakazakii* serotype O2 contains 28 defined sequence types [17]. The relatively small number of serotypes again indicates the possibility that the current RFLP and PCR-probe methods may not be robust enough to distinguish many DNA sequence variations. Serotype polymorphisms have been determined in Shiga-toxin producing *E. coli* following the sequencing of a 643 bp region of the *gnd* loci which is one of the O-antigen flanking genes [3]. Since >100 *Cronobacter* spp. genomes are available in the public domain, a similar sequence (allele) based serotyping approach using either or both of the O-antigen flanking genes can be considered which may overcome the current laborious approach requiring 15 primer pairs.

*Cronobacter* does produce EPS and forms biofilms on inert surfaces that are more resistant to cleaning and disinfectant agents [35–38]. Reported sources of *Cronobacter* in the hospital environment include infant formula preparation equipment, feeding bottles, and neonatal enteral feeding tubes [11, 34–37, 39]. The latter could act as loci for neonatal infection [35, 36].

In *Cronobacter* optimal EPS production is under nitrogen-limited growth conditions and is influenced by milk components [40, 41]. Consequently the organism has a notable extensive mucoid appearance on milk agar plates which causes the growth to drip on to the inverted lid [42]. The unique biophysical properties of this capsular material has led to patents being filed for its exploitation as a thickening agent in foods to replace xanthan gum [43]. Such capsular material production could also be important in desiccation persistence by the bacterium in the environment, in powdered infant formula (PIF), as well as in serum resistance and macrophage evasion. However, our understanding of the capsule is limited to a few studies on a limited number of strains, some of which were before the taxonomic revision of *Enterobacter sakazakii* to the genus *Cronobacter*.

The secreted EPS colanic acid is produced by some strains of *C. sakazakii* (ATCC 12868 and ATCC 29004) but not the species type strain ATCC 29544^T^ [43, 44]. It is composed of glucuronic acid (29–32), D-glucose (23–30), D-galactose (19-24), D-fucose (13–22) and D-mannose (0–0.8 %). Production of EPS from other named *Cronobacter* species has not been previously reported. Zogai et al. [9] used Calcofluor dye retention to demonstrate bacterial cellulose production by a commensal *Cronobacter* spp. strain (species not defined). Later, Lehner et al. [44] used fluorescence microscopy to demonstrate cellulose fibrils formation with a wide range of food and clinical *Cronobacter* spp. strains which at the time were only identified as *Enterobacter sakazakii*.

It is plausible that the strong association between *C. sakazakii* CC4 and neonatal meningitis is due to environmental fitness such as desiccation survival, resulting in their frequent isolation from both PIF and the environment of PIF manufacturing plants in Australia, China, Ireland, Switzerland and Germany [45–48]. Caubilla-Barron & Forsythe [49] reported the prolonged survival of desiccated *C. sakazakii* and especially capsulated strains in infant formula for over 2 years. This is considerably longer than *Salmonella enterica* and *Citrobacter koseri,* which were no longer recoverable after 14 and 8 months respectively.

Given such a wide range of environmental fitness and virulence related traits, it was deemed appropriate to characterise the clusters of capsule production related genes across the *Cronobacter* genus using the whole genome analysis of >100 strains. These genomes are available for analysis using the Bacterial Isolate Genome Sequence database (BIGSdb)-supported *Cronobacter* PubMLST open access database [17, 50].

## Results

### Diversity of *Cronobacter* O-antigen

The O-antigen polymerase and O-antigen flippase genes, *wzy* and *wzx* respectively, within the *rfb* locus have been the targets for molecular serotyping of *Cronobacter* species using PCR primers [23, 24]. Therefore they were selected for initial sequence variation and phylogenetic analysis of the O-antigen region. The extracted *wzx* and *wzy* sequences from Genbank as well as those from 104 genomes were aligned and phylogenetically analysed. The purpose of this analysis was to show the clustering of the predefined serotypes based on those presumed O-antigen specific sequences. However these genes were not found in all 104 strains of *Cronobacter. Wzy* was absent from the *C. sakazakii* O7 reference sequence (JQ674750), and *wzx* was absent from the genome of *C. condimenti* 1330^T^. Furthermore, genomic analysis revealed two novel O-antigen clusters for *C. muytjensii* which were defined as O3 and O4 (Additional file 1: Table S3). A potentially novel O-antigen region was also revealed in *C. dublinensis* strain 2030. However it is currently unclear if this matches the newly described *C. dublinensis* O3 as given by Blažková et al. [27] as the latter has been defined by RFLP restriction pattern and not DNA-sequence. Hence for the purpose of this study the O-antigen of *C. dublinensis* strain 2030 is referred to as ‘not assigned’ (na).

**Fig. 1:**
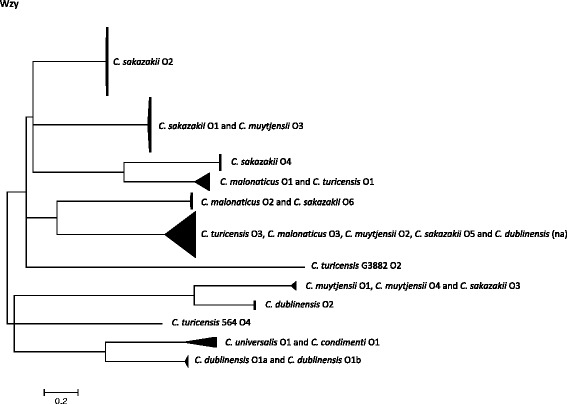
Phylogenetic tree of *wzy* sequences from across the *Cronobacter* genus. DNA sequences were aligned in MEGA version 5.2 using the ClustalW algorithm. The phylogenetic trees were generated using the Maximum Likelihood method

**Fig. 2:**
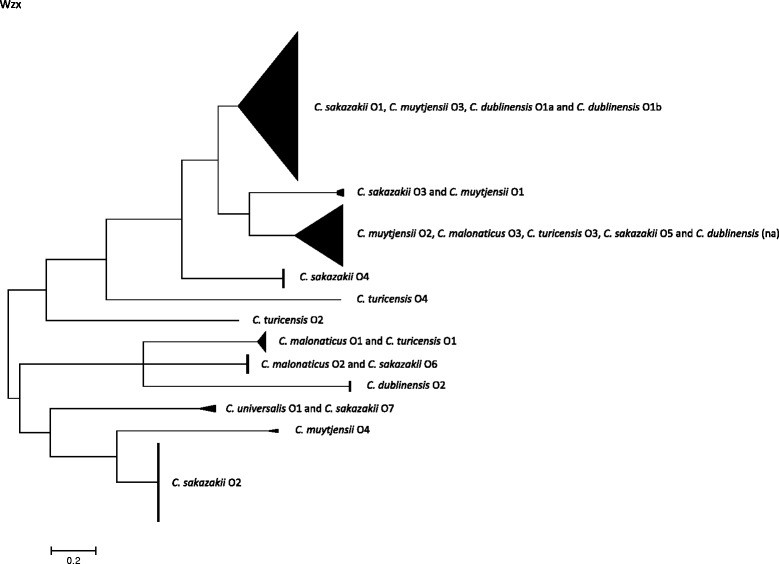
Phylogenetic tree of *wzx* sequences from across the *Cronobacter* genus. DNA sequences were aligned in MEGA version 5.2 using the ClustalW algorithm. The phylogenetic trees were generated using the Maximum Likelihood method

Phylogenetic trees of the whole genes *wzy* and *wzx* are shown in Figs. 1 and 2. The only corresponding serogroups which formed unique clusters were *C. sakazakii* serotypes O2, O4 and *C. dublinensis* O2; Figs. 1 and 2, Table 1.

**Table 1:** Clustering of *Cronobacter* O-serotypes according to w*zy, wzx, gnd* and *galF* sequence analysis

*Cronobacter* species	Serotype^a^	*gnd* alleles	*galF* alleles	*wzy* cluster group	*wzx* cluster group
*C. sakazakii*	O1	1,3,13,18,21,23	1,2,4,17,19,21	*C. muytjensii* O3^b^	*C. muytjensii* O3, *C. dublinensis* O1
	O2	2,13,19,28,29, 37	3,18,25,26	Unique cluster	Unique cluster
	O3	24	22	*C. muytjensii* O1	*C. muytjensii* O1
	O4	14	13	Unique cluster	Unique cluster
	O5	na	na	*C. turicensis* O3, *C. malonaticus* O3, *C. dublinensis* na^c^, *C. muytjensii* O2	*C. turicensis* O3, *C. malonaticus* O3, *C. dublinensis* na, *C. muytjensii* O2
	O6	na	na	*C. malonaticus* O2	*C. malonaticus* O2
	O7	na	na	Absent	*C. universalis* O1
*C. malonaticus*	O1	27, 33	6, 24	*C. turicensis* O1	*C. turicensis* O1
	O2	4, 26, 36	5, 23, 32	*C. sakazakii* O6	*C. sakazakii* O6
	O3	5, 38	6, 24	*C. sakazakii* O5, *C. turicensis* O3, *C. muytjensii* O2, *C. dublinensis* na	*C. turicensis* O3, *C. muytjensii* O2, *C. dublinensis* na
*C. turicensis*	O1	8	9, 28	*C. malonaticus* O1	*C. malonaticus* O1
	O2	na	na	Unique	Unique
	O3	22, 30, 34	20, 27, 30	*C. sakazakii* O5, *C. malonaticus* O3, *C. muytjensii* O2, *C. dublinensis* na	*C. sakazakii* O5, *C. malonaticus* O3, *C. muytjensii* O2, *C. dublinensis* na
	O4	6	7	Unique	Unique
*C. muytjensii*	O1	17	16	*C. sakazakii* O3	*C. sakazakii* O3
	O2	16	15	*C. sakazakii* O5, *C. turicensis* O3, *C. malonaticus* O3, *C. dublinensis* na	*C. malonaticus* O3, *C. turicensis* O3, *C. dublinensis* na
	O3	na	na	*C. sakazakii* O1	*C. sakazakii* O1, *C. dublinensis* O1
*C. dublinensis*	O1	12,15	12, 14	Unique cluster	*C. sakazakii* O1, *C. muytjensii* O3
	O2	11, 25, 32	11, 29	Unique cluster	Unique cluster
	na	35	31	*C. sakazakii* O5, *C. malonaticus* O3, *C. turicensis* O3, *C. muytjensii* O2	*C. malonaticus* O3, *C. turicensis* O3, *C. muytjensii* O2
*C. universalis*	O1	7	8	*C. condimenti* O1	*C. sakazakii* O7
*C. condimenti*	O1	9	33	*C. universalis* O1	Absent

^a^Serotypes as defined according to Jarvis et al. [23] and Sun et al. [24]

^b^Assigned in this paper

^c^Not assigned

The *wzy C. sakazakii* O2 cluster (*n* = 45) was composed of 7 sequence types; *C. sakazakii* ST3, ST4, ST13, ST15 (CC4), ST31, ST64, and ST218 (CC4). *C. sakazakii* O4 (*n* = 10) included one strain of *C. sakazakii* ST4, and all ST12, ST40 and ST45 strains. The *wzy* gene sequences of *C. sakazakii* serotypes O1 and O3 were indistinguishable from *C. muytjensii* O3 and, O1 and O4, respectively. *C. sakazakii* O5 clustered with *C. turicensis* O3, *C. malonaticus* O3, *C. dublinensis* na and *C. muytjensii* O2. *C. sakazakii* O6 was indistinguishable from *C. malonaticus* O2. *C. malonaticus* O1 and *C. turicensis* O1 were indistinguishable. *C. sakazakii* O7 could not be included in this analysis as the gene *wzy* is absent.

*C. malonaticus* O1 *wzy* cluster included two STs (ST60 & ST307), as well as the *C. turicensis* O1 (ST19). *C. malonaticus* O2 contained 8 strains from ST7, ST84, ST129 and ST302. The newly described *C. malonaticus* O3 cluster contained two *C. malonaticus* strains (ST11 and ST300) as well as those in *C. sakazakii* O5, *C. turicensis* O3, *C. muytjensii* O2 and *C. dublinensis* (na)*.*

According to the *wzy* gene sequence, as given above, *C. turicensis* serotype O1 clustered with *C. malonaticus* O1. *C. turicensis* O3 clustered with *C. malonaticus* O3, *C. muytjensii* O2, *C. sakazakii* O5 and *C. dublinensis* strain 2030 (serotype not assigned). *C. turicensis* O2 and O4 were composed of unique strains G3882 and 564, respectively.

As stated above, *C. muytjensii* O1, O4 and O3 clustered with *C. sakazakii* O3 and O1 respectively. *C. muytjensii* O2 clustered with serotypes in four other *Cronobacter* species; *C. sakazakii* O5, *C. malonaticus* O3, *C. turicensis* O3, and *C. dublinensis* (na).

*C. dublin*e*nsis* serotypes were split into two distinct clusters; Fig. 1. The *C. dublinensis* O1 cluster (containing the *C. dublinensis* subsp. *dublinensis* type strain and *C. dublinensis* subsp. *lartaridi*) was closer to the cluster of *C. universalis* O1 and *C. condimenti* O1. Whereas the *C. dublinensis* O2 cluster (containing *C. dublinensis* subsp. *lausanensis*) was close to the cluster of *C. sakazakii* O3 and *C. muytjensii* O1 and O4.

The clustering of *wzx* gene largely reflected that of *wzy*; Fig. 2, Table 1. The main differences were that *C. dublinensis* O1 clustered with *C. sakazakii* O1 and *C. muytjensii* O3, and that the *wzx* sequence of *C. sakazakii* O7 was indistinguishable from *C. universalis* O1.

Due to the non-phylogenetic congruence of the *wzy* and *wzx* sequences, the flanking genes *gnd* and *galF* were chosen to further investigate the diversity of the O-antigen region. A total of 38 unique alleles were observed at the *gnd* locus for the 104 *Cronobacter* strains. The *galF* locus generated slightly fewer (33) unique alleles. The additional *gnd* loci were primarily in *C. sakazakii* and included single nucleotide variants. The variant genes were assigned profile numbers and are recorded in the *Cronobacter* PubMLST database and given in Table 1. The allele length (501 bp) was chosen such that the region could easily be targeted by PCR primers in further laboratory analysis.

The detailed phylogenetic trees of *galF* and *gnd* are shown in Additional file 1: Figure S1 and S2. Nearly the same clustering as for the 38 *gnd* alleles was also observed with for the 33 *galF* alleles. Most *C. sakazakii* strains clustered together, with nearest neighbour *C. malonaticus*. The six *C. turicensis* strains all differed in their *gnd* sequences, and formed a cluster closely related to *C. universalis*. Similarly *C. muytjensii* and *C. dublinensis* clustered together with the *C. condimenti*. It was notable that the 10 *C. sakazakii* O4 strains formed a unique outgroup to the rest of the *Cronobacter* strains. This serotype is mostly composed of *C. sakazakii* ST12 strains.

The *galF* allele tree was similar to that of *gnd*; Additional file 1: Figure S1. *C. sakazakii* formed distinct clusters. Most were close relatives, whereas one was distant to most members of the genus. *C. malonaticus* strains formed two clusters, and branched close to the majority of *C. sakazakii* strains. The three strains of *C. muytjensii* formed a distinct cluster. These strains were *C. muytjensii* serotypes O1, O2 and the newly described O3 and O4. The *C. turicensis* strains all differed in their *galF* sequences, and formed a cluster closely related to *C. universalis*. The *C. dublinensis* strains were clustered into one group according to *gnd*, but two groups by *galF; C. dublinensis* subsp. *dublinensis* and *C. dublinensis* subsp*. lactaridi* were distant to *C. dublinensis* subsp. *lausanensis.* The latter group clustered with the *C. sakazakii* O4 cluster, and were distant from the rest of the *Cronobacter* genus; Additional file 1: Figure S1 and S2.

### Cellulose and enterobacterial common antigen diversity

The cellulose gene cluster composed of 9 genes (*bcsCZBAQEFG* and *yhjR*) was found in nearly all *Cronobacter* strains. The exceptions were all (*n* = 7) *C. sakazakii* ST13 and CC100 strains and the single strain of *C. condimenti* 1330^T^. These were confirmed using Calcofluor agar plates (data not presented).

**Fig. 3:**
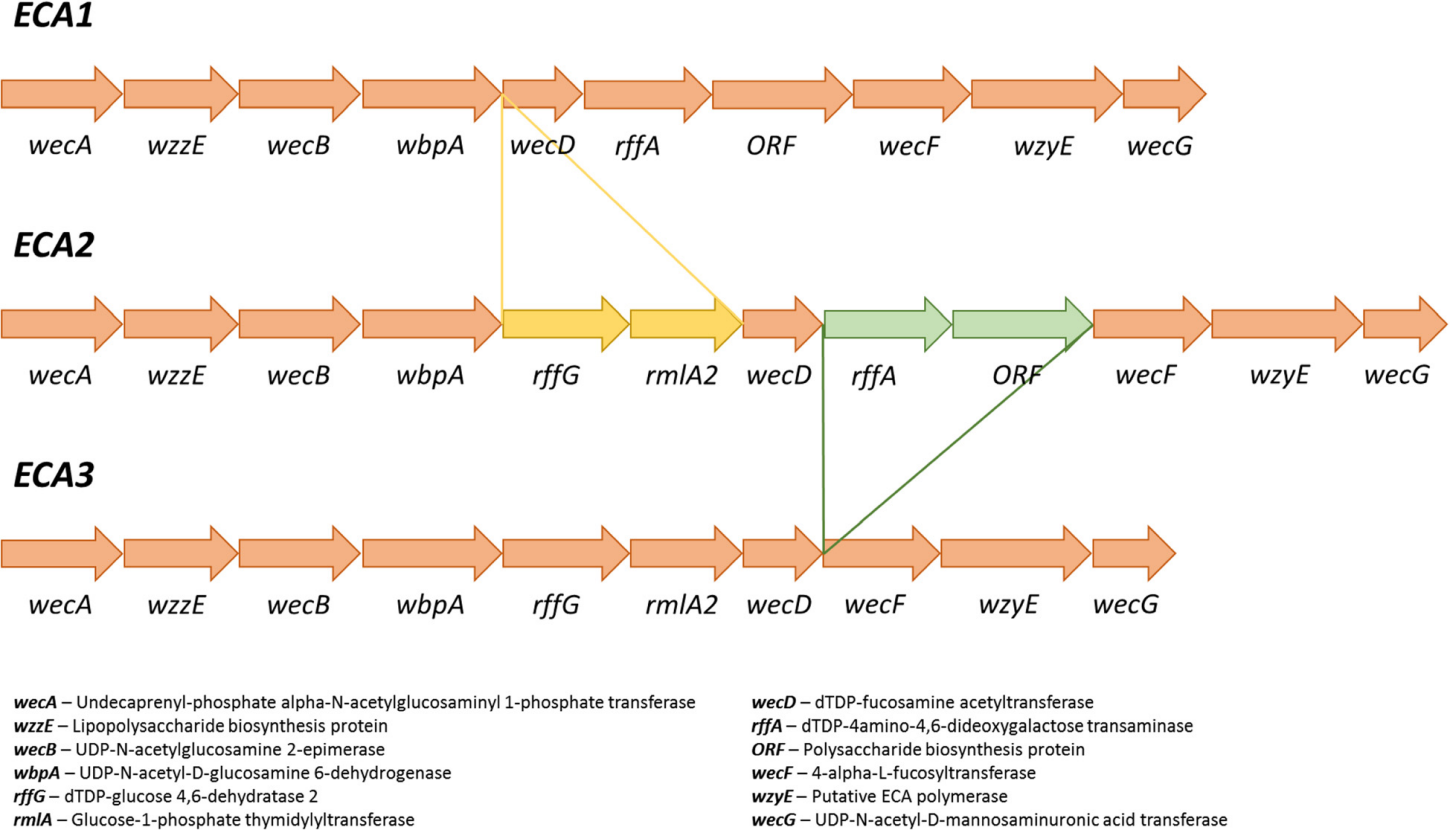
Comparison of enterobacterial common antigen (ECA) gene clusters across the *Cronobacte*r genus

The enterobacterial common antigen (ECA) gene cluster composed of 10–12 genes was found in all *Cronobacter* strains; Fig. 3, Table 2. There were three variants; ECA1 in all *C. sakazakii* and *C. malonaticus* 507, made up of 10 genes, missing *rffG* and *rmlA2*, and ECA2 in nearly all other species and strains, made up of 12 genes. Other variations were the absence of *rffA* and ORF1 in *C. muytjensii* strain 530 which was defined as ECA3, and a truncated *rmlA2* in *C. turicensis* strains 1552 and 1880, these were still assigned as ECA2.

**Table 2:** Serotype and capsular profiles of *Cronobacter* species

Species	Number of strains	Sequence type	Clonal complex	*gnd* allele	*galF* allele	O-type	K-antigen type	Colanic acid type	Cellulose *bcs* genes	Enterobacterial common antigen type
*C. sakazakii*	5	1	1	1	2	O1	K1	CA1	+	ECA1
*C. sakazakii*	1	14	1	1	2	O1	K1	CA1	+	ECA1
*C. sakazakii*	5	8	8	3	4	O1	K1	CA1	+	ECA1
*C. sakazakii*	1	64	64	37	26	O2	K1	CA2	+	ECA1
*C. sakazakii*	1	12		24	22	O3	K2	CA1	+	ECA1
*C. sakazakii*	1	31	31	29	26	O2	K2	CA2	+	ECA1
*C. sakazakii*	2	3	3	20	18	O2	K2	CA2	+	ECA1
*C. sakazakii*	34	4	4	2	3	O2	K2	CA2	+	ECA1
*C. sakazakii*	4	15	4	2	3	O2	K2	CA2	+	ECA1
*C. sakazakii*	1	218	4	2	3	O2	K2	CA2	+	ECA1
*C. sakazakii*	1	4	4	14	13	O4	K2	CA2	+	ECA1
*C. sakazakii*	7	12		14	13	O4	K2	CA2	+	ECA1
*C. sakazakii*	2	40	45	14	13	O4	K2	CA2	+	ECA1
*C. sakazakii*	5	13	13	28	25	O2	K2	CA2	CL-	ECA1
*C. sakazakii*	1	100	100	13	1	O1	K2	CA1	CL-	ECA1
*C. sakazakii*	1	125	100	13	1	O1	K2	CA1	CL-	ECA1
*C. sakazakii*	1	148	16	23	21	O1	K2	CA1	+	ECA1
*C. sakazakii*	1	257	21	21	19	O1	K2	CA1	+	ECA1
*C. sakazakii*	1	297		18	17	O1	K2	CA1	+	ECA1
*C. malonaticus*	1	307	112	33	6	O1	K2	CA2	+	ECA2
*C. malonaticus*	2	60		27	24	O1	K2	CA2	+	ECA2
*C. malonaticus*	4	7	7	4	5	O2	K1	CA1	+	ECA2
*C. malonaticus*	1	84	7	4	5	O2	K1	CA1	+	ECA2
*C. malonaticus*	2	302		36	32	O2	K1	CA1	+	ECA2
*C. malonaticus*	1	129	129	26	23	O2	K1	CA1	+	ECA2
*C. malonaticus*	1	11		5	6	O3	K1	CA2	+	ECA1
*C. malonaticus*	1	300	300	38	24	O3	K1	CA2	+	ECA2
*C. turicensis*	1	19	24	8	9	O1	K1	CA2	+	ECA2
*C. turicensis*	1	5		6	7	O4	K1	CA2	+	ECA2
*C. turicensis*	1	344		34	30	O3	K1	CA2	+	ECA2
*C. turicensis*	1	72	72	30	27	O3	K1	CA2	+	ECA2
*C. turicensis*	1	35		22	20	O3	K2	CA2	+	ECA2
*C. turicensis*	1	342		31	28	O1	K2	CA2	+	ECA2
*C. dublinensis* sbsp. *dublinensis*	1	106		12	12	O1b	K1	CA1	+	ECA2
*C. dublinensis* subsp. *lactaridi*	1	79		15	14	O1a	K1	CA1	+	ECA2
*C. dublinensis* sbsp. *lausanensis*	2	80	80	11	11	O2	K1	CA2	+	ECA2
*C. dublinensis*	1	36	80	11	11	O2	K1	CA2	+	ECA2
*C. dublinensis*	1	346		25	11	O2	K1	CA2	+	ECA2
*C. dublinensis*	1	341		32	29	O2	K1	CA2	+	ECA2
*C. dublinensis*	1	301		35	31	na	K2	CA2	+	ECA2
*C. muytjensii*	1	347		10	10	O3	K1	CA1	+	ECA2
*C. muytjensii*	1	81	81	16	15	O2	K1	CA2	+	ECA2
*C. muytjensii*	1	294		17	16	O1	K1	CA1	+	ECA3
*C. universalis*	1	54		7	8	O1	K1	CA2	+	ECA2
*C. condimenti*	1	98		9	33	O1	K1	CA2	CL-	ECA2

### Colanic acid gene cluster diversity

The colanic acid encoding gene cluster (CA) was located adjacent to the O-antigen region and separated by *galF*. Only two variants were found, CA1 and CA2 composed of 21 and 20 genes respectively, which differ in the presence of *galE* (encoding for UDP-N-acetyl glucosamine 4-epimerase) in CA1, and absence in CA2; Table 2. The occurrence of these two capsular regions varied across the genus. CA1 was found in most *C. sakazakii* sequence types, and other species. Whereas, CA2 was primarily found in *C. sakazakii* sequence types ST4 and ST12. CA2 was also found in only a few of the 14 *C. malonaticus* strains; 507 (ST11) and 1569 (ST307), 1846, 687 (ST60) and 2109 (ST300). CA2 was not found in any *C. malonaticus* ST7 strains (*n* = 5).

### K-antigen characterisation

**Fig. 4:**
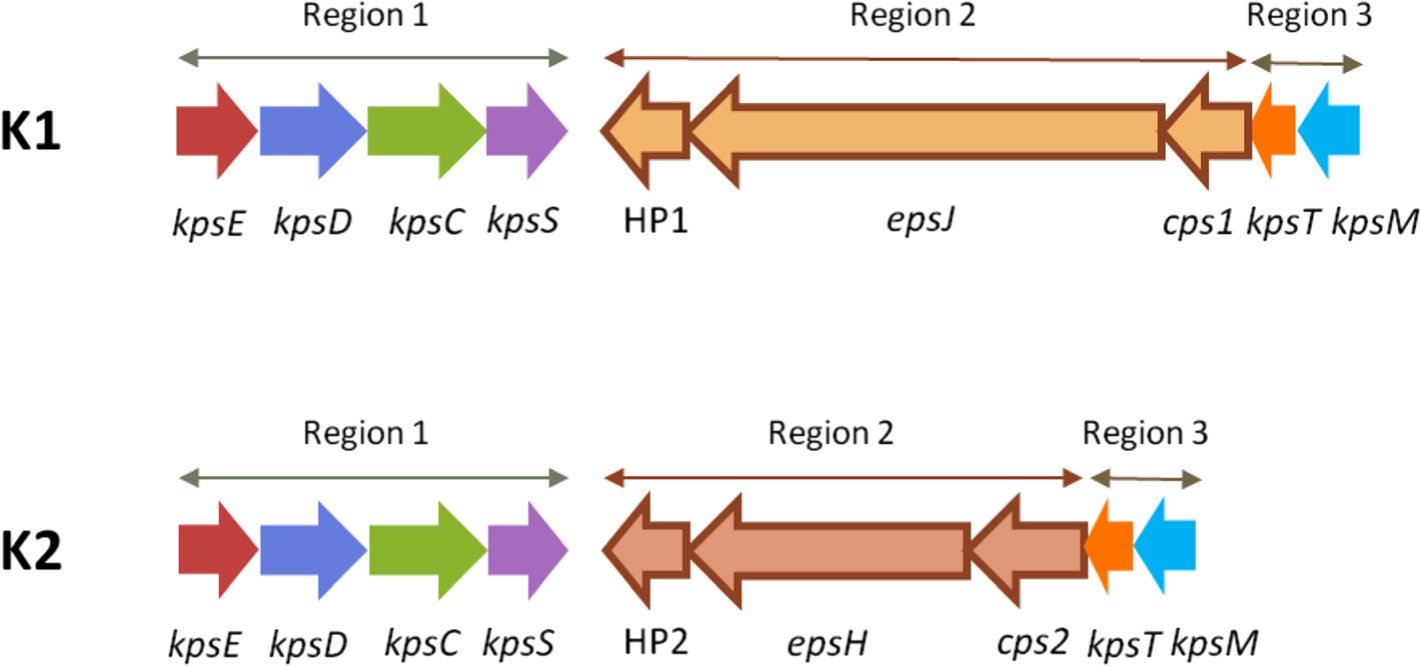
*Cronobacter* spp. K1 and K2 Region 1–3 *kps* genes

A previously uncharacterised capsular region (*kps*) was found in all (*n* = 104) *Cronobacter* strains and species. The region was homologous to the previously well described K-antigen gene cluster of *E. coli,* and which is composed of three regions. The K-antigen Region 1 (*kpsEDCS*) and Region 3 (*kpsTM*) were conserved across the genus. There were two variants of Region 2. Both variants encoded for genes for which no specific nearest matches (<50 % similarity) to specifically identified genes could be found in any BLAST search. Therefore the open reading frames are described here as either hypothetical proteins or by the general term glycosyltransferase; Table 3 and Fig. 4. The two glycosyltransferases (I and II) in Region 2 were not identical and differed in their length and GC% content; see Table 2. Region 2 GC% content of *kps* capsule type 1 was 42.8–46.8 %, whereas that of *kps* capsule type 2 was notably lower at 32.4–34.7 %. The GC content of Regions 1 and 3 was between 50–63 %. Comparison of *kpsS* (encoding for the capsular polysaccharide transport protein) showed there was sequence variation according to *kps* Region 2.

**Table 3:** *Cronobacter kps* region 2 genes

*Kps* capsule type	*Kps* region 2 gene	Function	Size (kb)	GC content (%)
1	hyp1	Hypothetical protein	1.23	44.9
	*espJ*	Glycosyltransferase I	3.78	42.8
	*cps1*	Capsular polysaccharide phosphotransferase	1.11	46.8
2	hyp2	Hypothetical protein	1.17	32.4
	*epsH*	Glycosyltransferase II	2.28	34.7
	*cps2*	Capsular polysaccharide phosphotransferase	1.09	33.8

**Fig. 5:**
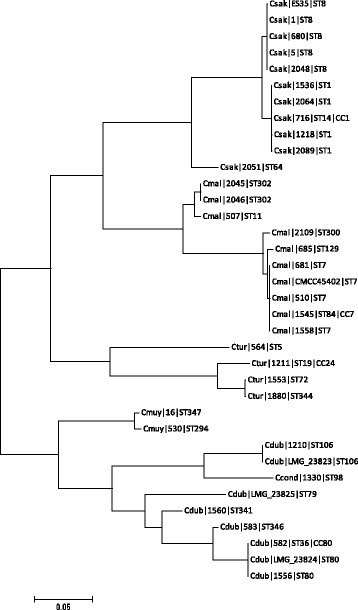
Phylogenetic tree of *Cronobacter* type 1 KPS Region 2 glycosyltransferase

*Cronobacter* strains with K-antigen type 1 were in all seven *Cronobacter* species. This included *C. sakazakii* sequence types ST1, ST8, ST14, ST64, *C. malonaticus* ST7, ST11, ST84, ST300, ST302, *C. turicensis* ST5, ST19, ST72, and ST344, all *C. dublinensis* strains except ST301, and all strains of *C. condimenti*, *C. muytjensii* and *C. universalis*; Table 2. Phylogenetic analysis of the glycosyltransferase I region largely reflected the whole genome phylogeny of the genus, with *C. sakazakii* and *C. malonaticus* clustering separately from *C. turicensis*, and *C. dublinensis* and *C. muytjensii* forming separate clusters; Fig. 5. Both *C. malonaticus* and *C. dublinensis* clusters separated into two smaller groups, reflecting the sequence types.

**Fig. 6:**
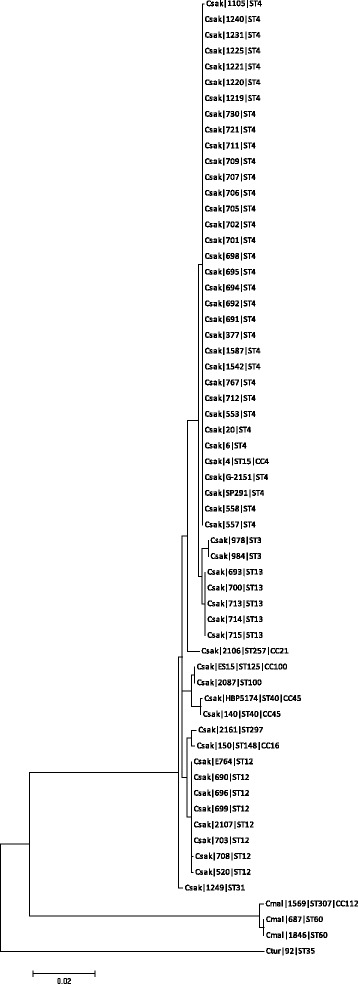
Phylogenetic tree of *Cronobacter* type 2 KPS Region 2 glycosyltransferase

The occurrence of K-antigen type 2 in the *Cronobacter* genus was more limited than type 1; Table 2. It was only found in *C. sakazakii*, *C. malonaticus* and *C. turicensis*. The main *C. sakazakii* sequences types with K-antigen type 2 were ST3, ST12, ST13, and those in clonal complex of ST4. The K-antigen type 2 was also found in *C. malonaticus* ST60 & ST307, *C. turicensis* ST35 & ST342 (both sialic acid utilizers), and *C. dublinensis* ST301. Phylogenetic analysis showed the *C. sakazakii* strains formed one large cluster, separate from the remaining species; Fig. 6.

**Fig. 7:**
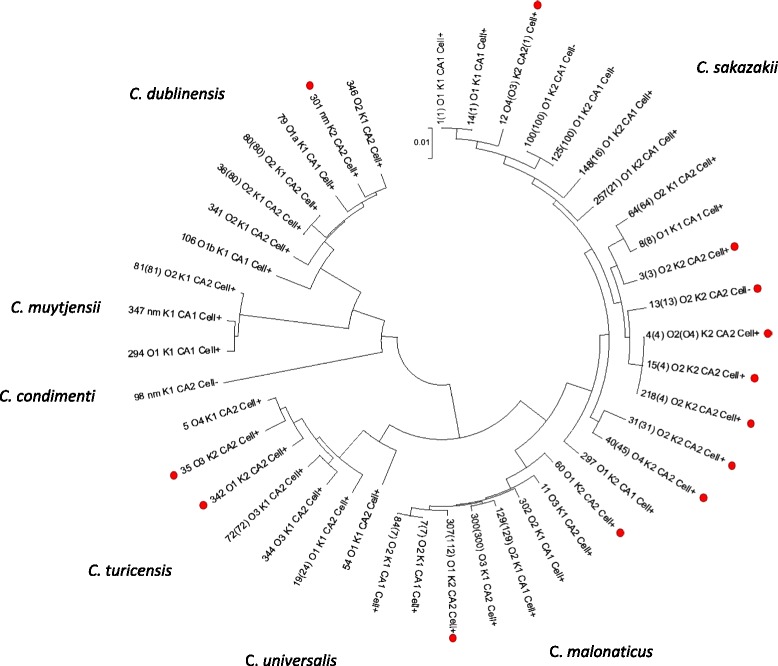
7-loci phylogenetic tree of *Cronobacter* genus with capsular gene profile K2:CA2 indicated (•)

Collating the occurrence of capsular genes and isolate source detail revealed a pattern in the presence of three capsule gene clusters; K-antigen, colanic acid and cellulose biosynthesis genes. This is summarised in Table 2, and for each strain in Additional file 1: Table S4. The possession of these three genes clusters was not phylogenetically linked; Fig. 7. Strains which were K2:CA2:Cell^+^ were sequence types of *C. sakazakii* and *C. malonaticus* which were mostly associated with cases of neonatal meningitis and necrotising enterocolitis. These included all *C. sakazakii* ST3, ST12 and strains in the clonal lineage ST4, as well as *C. malonaticus* ST60 and ST307. *C. turicensis* ST35 and ST342 also had the capsular profile K2:CA2:Cell+. It should be noted that *C. turicensis* strains in these two STs are able to utilise sialic acid as carbon source for growth, a trait otherwise only found in *C. sakazakii*. Strains with the capsular profile K2:CA2 but lacking the cellulose biosynthesis genes *bcs*, were all in *C. sakazakii* ST13, and had not been isolated from serious neonatal infections. Instead they had been isolated from prepared infant formula or from stool samples of neonates with minor digestive problems or asymptomatic.

## Discussion

It is well recognised that bacterial cell surface structures can have a major role in pathogenicity, and can be used for typing schemes. Molecular methods for the characterisation and identification of O-antigen determinants of Gram-negative bacteria have been devised using RFLP profiling of the *rfb* locus as well as allele-specific PCR primers [23, 24]. The entire O-antigen encoding gene cluster can be amplified using primers that target conserved regions in the neighbouring *gnd* sequence and JUMPstart sequence, and enzymic digestion of this amplicon can identify RFLPs correlating to O-antigen determinants [51]. This approach was initially applied to *Cronobacter* by Mullane et al. [22] and expanded by other groups [23, 24]. This has led to 24 O-antigen serotypes being described across the *Cronobacter* genus [27]. However this approach is less discriminatory than the 7-loci MLST scheme which has >350 defined types [17]. This is probably due to several reasons. First, the targeting of genes (*wzx* and *wzy*) which are largely conserved across the genus. Second, the lack of accuracy when using gel electrophoresis of RFLP products in recognising DNA-sequence variants. This is because unique restriction profiles may not be present for all serotypes or the size of the PCR amplicons cannot be accurately resolved. These issues have previously been identified with O-serotyping of Shiga-toxigenic *E. coli* (STEC) [3].

Phylogenetic analysis based on *wzx* and *wzy* sequence analysis of the *Cronobacter* O-antigen reveals the extent to which these gene sequences are indistinguishable across different *Cronobacter* species; Figs. 1 and 2. The only serotypes with unique sequences within a species were *C. sakazakii* O2, O4 and *C. dublinensis* O2. In contrast some gene sequences, such as *C. sakazakii* O5, were indistinguishable across 5 *Cronobacter* species; Table 1. Some overlap of *wzx* and *wzy* genes across the *Cronobacter* genus has been reported before, but the extent of the issue has been more fully revealed here.

The lack of phylogeny congruence in the O-antigen has resulted in the need for prior accurate speciation of *Cronobacter* isolates. Although PCR probes *rpoB* and *cgcA* have been advocated to speciate presumptive *Cronobacter* isolates, this is no longer recommended since it is now apparent that neither method is reliable. Jackson et al. [33, 34] have reported the mis-identification of *Cronobacter* and non-*Cronobacter* strains using these methods, and also a previously reported outbreak of *C. sakazakii* based on *rpoB* PCR probe identification was re-investigated and found to be due to *Enterobacter* spp. and *E. hormaechei* instead. An alternative use of *fusA* allele sequencing which corresponds to whole genome phylogeny is considerably more reliable and is one of the standard 7-loci for *Cronobacter* MLST [14, 19]. The latter is supported by an open access database of >1000 strains and >100 whole sequenced genomes [17].

As an alternative to RFLP for serotyping STEC, Gilmour et al. [3] successfully applied *gnd* sequence polymorphism analysis. Consequently, the sequence polymorphisms in the O-antigen flanking genes *galF* and *gnd* were evaluated for *Cronobacter* strain typing. Similarly in *Salmonella* the O-antigen encoding *rfb* locus flanking gene *gnd* maintains high levels of polymorphism linked to the diverse *rfb* operon [52]. These genes follow more closely the phylogeny of the genus than *wzx* and *wzy*. The exceptions being certain sequence types of *C. sakazakii* and *C. dublinensis,* which are discussed later. This study revealed 33 and 38 distinguishable variants, respectively; Table 2, Additional file 1: Figure S1 and S2. Unfortunately not all previously published serotypes could be assigned *gnd* or *galF* alleles as the region has not been sequenced by previous researchers investigating this particular region, and no appropriate whole genomes have been released. Despite the reported lack of relationship between O-antigen and the genus phylogeny [25, 26], this study has shown the closer correlation in sequence of the O-antigen flanking genes with the phylogenetic structure of the genus as defined using whole genome sequences which correlates with sequence typing [14, 19]. It is proposed that each defined *gnd* or *galF* allele could correspond to a discrete O-PS structure, and this warrants further investigation.

*C. sakazakii* ST12 strains were serotype *C. sakazakii* O4 and formed a separate cluster outgroup with *C. dublinensis* ST106 from the other *Cronobacter* with both *galF* and *gnd* phylogenetic trees; Additional file 1: Figure S1 and S2. This group corresponds with those identified by Sun et al. [26] and Shashkov et al. [32] regarding their similarity to *E. coli* O103.

Strains in *C. dublinensis* were in two cluster groups with *gnd*, but not *galF*. Using PubMLST.org/cronobacter/ genome comparator and COG-MLST tools [17] it was noted that *C. dublinensis* ST106 strains in the first cluster group (nearest neighbours *C. turicensis* and *C. muytjensii*) are able to utilise malonate. In contrast, the outlier group of *C. dublinensis* O2 with nearest neighbour *C. sakazakii* ST12 do not metabolise malonate (laboratory studies data not presented). This curious distribution of genes may reflect gene loss or gain during the genus evolution and adaption.

As previously reported, *C. sakazakii* does not possess curli fimbriae [14]. However these are encoded in some *C. malonaticus*, *C. turicensis* and *C. universalis* strains. The reason for this variation is unknown, but is surprising given curli fimbriae are associated with bacterial resistance to bile salts, and *C. sakazakii* is an enteric pathogen. Passage through the digestive tract will expose *Cronobacter* cells to high concentrations of bile salts which could damage the cell membrane. The organism does grow in *Enterobacteriaceae* Enrichment broth and on standard *Enterobacteriaceae* isolation media such as VRBGA. These contain 0.3 and 0.15 % bile salts respectively, and therefore the organism possesses other resistance mechanisms to bile salts.

The ECA is important for the bacterial cell envelope integrity, flagellum expression, and resistance to bile salts [53]. It is plausible that ECA contributes to *Cronobacter* enteric survival by protecting the organism from bile salts, as has been proposed for *Salmonella*. The gene *wecD* is associated with bile salts resistance in *Salmonella* and was present in the three ECA variants. It is currently unknown what the biochemical and functional significance are for the differences in the three ECA gene clusters in *Cronobacter.*

The colanic acid gene cluster CA2 was primarily found in *C. sakazakii* sequence types ST4 and ST12. These are clinically important sequence types with respect to neonatal infections. CA2 was also found in five of the 14 *C. malonaticus* strains; 507 (ST11) and 1569 (ST307), 1846, 687 (ST60) and 2109 (ST300). CA2 was not in any *C. malonaticus* ST7 strains (*n* = 5), which is the sequence type predominantly associated with adult infections. The significance of this variation is discussed further with respect to the K-antigen.

Genes involved in CPS biosynthesis and transport are typically organised within a so-called capsular gene cluster. CPS transport genes are generally well conserved. The Group 2 K-antigen assembly system of *E. coli* has been well studied both genetically and biochemically as an ABC-transporter-dependent pathway [1, 2]. The conserved Regions 1 (*kpsFEDUCS*) and 3 (*kpsTM*) encode for the poly-KDO linker and transport proteins for initiation of chain elongation and translocation to the cell surface. Region 2 genes encode for the glycosyltransferases and other enzymes responsible for biosynthesis of the K-antigen-specific CPS. In comparison with Groups 1, 3, and 4, expression of *E. coli* Group 2 CPS is subject to thermoregulation (<20 °C and 37 °C) [10].

In their genomic analysis of 11 *Cronobacter* strains, Joseph et al. [14] were the first to note that the organism possessed a sequence type variable capsular polysaccharide encoding region (ESA_03350-59) but did not undertake any further analysis. This new study shows this uncharacterised capsular region is found in all (*n* = 104) *Cronobacter* strains and species. The region was homologous to the well described K-antigen gene cluster from *E. coli* and is composed of three regions. The K-antigen Region 1 (*kpsEDCS*) and Region 3 (*kpsTM*) were conserved across the genus. However, there were two variants of Region 2. Both encode for genes for which no specific nearest matches (<50 % similarity) to identifiable genes could be found in any BLAST search; Fig. 4. The glycosyltransferases genes in Region 2 differed in their length and CG % content. Presumably this reflects differences in the polysaccharide which is synthesized and exported to the cell surface, and corresponds with the differences in the *kpsS* sequence. The GC % content of both Region 2 gene clusters were lower than the average GC % of *Cronobacter* (56 %); see Table 2. The composition of the K-antigen-specific CPS is currently unknown, but could be an important virulence or environmental fitness trait. The chemical structure of the K-antigen will greatly assist in elucidating the function of the genes in Region 2.

All strains (*n* = 54) of *C. sakazakii* CC4 and ST12 strains had the capsular profile K2:CA2:Cell^+^. These sequence types are strongly associated with severe neonatal infections (meningitis and NEC) [13, 17–19, 54]. However strains belonging to other STs may also cause severe neonatal infection. *C. sakazakii* ST31 strain 1249 was a CSF isolate from a severe case of bacterial meningitis in the UK. This strain had the capsular profile K2:CA2:Cell^+^. It is also of particular interest that *C. malonaticus* ST307 strain 1569 has the capsular profile K2:CA2:Cell^+^. Thus it has the same capsular profile as *C. sakazakii* CC4, the *Cronobacter* pathovar for neonatal meningitis. This may be highly significant since strain 1569 it is the only recorded isolate from a fatal meningitis neonatal case due to *C. malonaticus* [54]. In contrast, *C. malonaticus* ST7 is the most frequently isolated *C. malonaticus* sequence type, and is associated with adult infections [17, 55, 56]. As shown in Table 2, *C. malonaticus* ST7 strains have the capsular profile K1:CA1:Cell^+^; Fig. 7. *C. turicensis* strains in ST35 and ST342 also had the capsular profile K2:CA2:Cell^+^. Although these specific strains are non-clinical in original, it is of interest that these strains are able to utilize sialic acid as a carbon source, an ability which is otherwise limited to *C. sakazakii.* Such metabolism could be significant virulence trait given the sugars occurrence in breast milk, infant formula, mucin and gangliosides [57].

*C. sakazakii* ST13 strains had the profile K2:CA2:Cell^−^, since they lack the cellulose biosynthesis *bcs* genes. These strains had been isolated from the stools of two infants and three prepared infant formula feeds during a NICU *C. sakazakii* ST4 outbreak, but no severe infections were reported in these particular infants other than a minor digestive problem in one of them [42]. Further research is required to elucidate the possible roll of bacterial cellulose with pathogenesis. *C. sakazakii* ST64 strains are K1:CA2:Cell+, varying from the neonatal associated capsular profile by the K-antigen. This sequence type is not associated with clinical cases, and yet has been often isolated from powdered infant formula and the environment of manufacturing plants in Switzerland, China, France, Czech Republic, and Germany [17, 45–48]. Therefore it is plausible that the possession of K2 and CA2 may give combined favourable phenotype for desiccation persistence and virulence such as serum resistance of macrophage survival, resulting in an increased risk of neonatal infection.

Phylogenetic analysis based on the sequence type (3036 nt concatenated length) for the occurrence of the capsule gene clusters for the K-antigen, colanic acid and cellulose biosynthesis showed the capsular profile of K2:CA2:Cell^+^ was not phylogenetically localised. While there was an expected high number of *C. sakazakii* clonal complex 4 STs with the trait, due to over-representation in whole genome sequenced strains, the capsular profile was also found in distantly related *C. sakazakii* sequence types (ie. ST12, ST31 & ST45), as well as *C. malonaticus, C. turicensis*, and *C. dublinensis*. These three species are frequently isolated from clinical cases of *Cronobacter* infection. The proposed capsular profiling of *Cronobacter* may direct new understanding of the environmental persistence and virulence traits in this organism, and warrants further investigation with respect to chemical analysis of capsular material and a more robust DNA sequence-based typing scheme which harmonises previous conflicting O-antigen analysis.

## Methods

### Bacterial strains

A total of 104 genomes were analysed for this study, which were the total number of genomes available (July 2015). Relevant information such as sequence type, source and year of isolation is given in Additional file 1: Table S1. Additional metadata can be obtain from the open access *Cronobacter* PubMLST database; http://pubmlst.org/cronobacter/.

### DNA sequences

Whole genome DNA sequences collated at http://pubmlst.org/cronobacter/ were investigated. *In silico* analyses were carried out using search options, such as BLAST, on the *Cronobacter* PubMLST portal accessible at: http://pubmlst.org/perl/bigsdb/bigsdb.pl?db=pubmlst_cronobacter_isolates.

### O-antigen gene regions

For comparative purposes, published *Cronobacter* O-antigen sequences were downloaded from Genbank; Additional file 1: Table S2.

### *Gnd* and *galF* allele allocation

DNA sequences of *gnd* (501 bp, positions 114–614) and *galF* (501 bp, positions 376–876) were assigned novel alleles numbers using the *Cronobacter* PubMLST database by its curator (SJF).

### DNA annotation and visualisation tools

Bacterial DNA sequences were investigated using the genome browser and annotation tool Artemis [58].

#### Phylogenetic analysis

DNA sequences were carefully curated prior to and after alignment and phylogenetic analyses in order to maximise the quality of the results using the satisfactory default parameters for the latter analyses. DNA sequences were aligned in MEGA version 5.2 using the ClustalW algorithm [59] set to default parameters settings.

The phylogenetic trees were generated using the Maximum Likelihood (ML) method based on the Tamura-Nei model with the additional parameters set to default settings. All phylogenetic trees are drawn to scale with branch lengths measured in the number of substitutions per site.

#### Ethics statement

All clinical data are taken from a previous publications associated with the sequenced bacterial strains.

## Additional file

Additional file 1: Table S1. Summary of strains with source details from *Cronobacter* PubMLST database. **Table S2.** Genbank accession numbers used for O-antigen loci. **Table S3.** Description of *C. muytjensii* O-antigen designations. **Table S4.** Serotype and capsular profiles of *Cronobacter* species. **Figure S1.** Phylogenetic tree of *Cronobacter* spp. *galF* sequences (total length 501 bp). **Figure S2.** Phylogenetic tree of *Cronobacter* spp. *gnd* sequences (total length 501 bp). (DOC 292 kb)

## Abbreviations

BIGSdb: Bacterial Isolate Genome Sequence database
CC: Clonal complex
CPS: Capsular polysaccharides
ECA: Enterobacterial common antigen
EPS: Exopolysaccharides
MLST: Multilocus sequence typing
NICU: Neonatal intensive care unit
NM: Non-motile
PCR: Polymerase chain reactions
PIF: Powdered infant formula
RFLP: Random fragment length polymorphism
ST: Sequence type
STEC: Shiga-toxigenic *E. coli*
