# Supplementary material for: Capsular profiling of the Cronobacter genus and the association of specific Cronobacter sakazakii and C. malonaticus capsule types with neonatal meningitis and necrotizing enterocolitis
Source: BMC Genomics. 2015 Oct 8;16:758. doi: 10.1186/s12864-015-1960-z (PMC4599207; doi:10.1186/s12864-015-1960-z)
Supplement: Additional file 1: Table S1. — Summary of strains with source details from Cronobacter PubMLST database. Table S2. Genbank accession numbers used for O-antigen loci. Table S3. Description of C. muytjensii O-antigen designations. Table S4. Serotype and capsular profiles of Cronobacter species. Figure S1. Phylogenetic tree of Cronobacter spp. galF sequences (total length 501 bp). Figure S2. Phylogenetic tree of Cronobacter spp. gnd sequences (total length 501 bp). (DOC 292 kb) [file 12864_2015_1960_MOESM1_ESM.doc]

Supplementary files

Table S1: Summary of strains with source details from *Cronobacter* PubMLST database

| **Species** | **Isolate** | **Country** | **Source** | **Year** |
| --- | --- | --- | --- | --- |
| *C. sakazakii* | 658 (BAA-894) | USA | Formula | 2001 |
| *C. sakazakii* | 1218 | USA | Clinical | 2001 |
| *C. sakazakii* | 1536 | Germany | Environment | 2009 |
| *C. sakazakii* | 2064 | France | Environmental |  |
| *C. sakazakii* | 2089 | France | Clinical | 2004 |
| *C. sakazakii* | 716 | France | Infant formula | 1994 |
| *C. sakazakii* | ATCC29544^T^ | USA | Clinical | 1980 |
| *C. sakazakii* | 5 | Canada | Clinical | 1990 |
| *C. sakazakii* | 2048 | France | Environmental |  |
| *C. sakazakii* | 680 | USA | Clinical | 1977 |
| *C. sakazakii* | ES35 | Israel | Clinical |  |
| *C. sakazakii* | 2051 | France | Environmental |  |
| *C. sakazakii* | 520 | Czech Republic | Clinical | 1983 |
| *C. sakazakii* | 1249 | UK | Clinical | 2010 |
| *C. sakazakii* | 978 | UK | Clinical | 2007 |
| *C. sakazakii* | 984 | UK | Clinical | 2007 |
| *C. sakazakii* | 377 | UK | Milk powder | 1950 |
| *C. sakazakii* | 6 | Canada | Clinical | 1990 |
| *C. sakazakii* | 20 | Czech Republic | Clinical | 2003 |
| *C. sakazakii* | 553 | Netherlands | Clinical | 1977 |
| *C. sakazakii* | 557 | Netherlands | Clinical | 1979 |
| *C. sakazakii* | 1533 | Germany | Environment | 2006 |
| *C. sakazakii* | 1537 | Germany | Environment | 2009 |
| *C. sakazakii* | 1542 | Germany | Environment | 2009 |
| *C. sakazakii* | 1587 | Israel | Clinical | 2000 |
| *C. sakazakii* | 691 | France | Clinical | 1994 |
| *C. sakazakii* | 692 | France | Clinical | 1994 |
| *C. sakazakii* | 694 | France | Clinical | 1994 |
| *C. sakazakii* | 695 | France | Clinical | 1994 |
| *C. sakazakii* | 698 | France | Clinical | 1994 |
| *C. sakazakii* | 701 | France | Clinical | 1994 |
| *C. sakazakii* | 702 | France | Clinical | 1994 |
| *C. sakazakii* | 705 | France | Clinical | 1994 |
| *C. sakazakii* | 706 | France | Clinical | 1994 |
| *C. sakazakii* | 707 | France | Clinical | 1994 |
| *C. sakazakii* | 709 | France | Clinical | 1994 |
| *C. sakazakii* | 711 | France | Clinical | 1994 |
| *C. sakazakii* | 712 | France | Prepared formula | 1994 |
| *C. sakazakii* | 767 | France | Clinical | 1994 |
| *C. sakazakii* | 1105 | UK | Weaning food | 2008 |
| *C. sakazakii* | 721 | USA | Clinical | 2003 |
| *C. sakazakii* | 730 | France | Clinical | 1994 |
| *C. sakazakii* | 1219 | USA | Clinical | 2009 |
| *C. sakazakii* | 1220 | USA | Clinical | 2003 |
| *C. sakazakii* | 1221 | USA | Clinical | 2003 |
| *C. sakazakii* | 1225 | USA | Clinical | 2007 |
| *C. sakazakii* | 1231 | New Zealand | Clinical | 2005 |
| *C. sakazakii* | 558 | Netherlands | Clinical | 1983 |
| *C. sakazakii* | SP291 | Ireland | Infant formula factory |  |
| *C. sakazakii* | G-2151 | USA | Clinical |  |
| *C. sakazakii* | 4 | Canada | Clinical | 1990 |
| *C. sakazakii* | ES713 | USA | Infant formula |  |
| *C. sakazakii* | 1240 (CDC 2009-06-01) | USA | Clinical | 2009 |
| *C. sakazakii* | 690 | France | Clinical | 1994 |
| *C. sakazakii* | 696 | France | Clinical | 1994 |
| *C. sakazakii* | 699 | France | Clinical | 1994 |
| *C. sakazakii* | 708 | France | Clinical | 1994 |
| *C. sakazakii* | 2107 | Belgium | Clinical |  |
| *C. sakazakii* | 703 | France | Clinical | 1994 |
| *C. sakazakii* | E764 | USA | Clinical |  |
| *C. sakazakii* | 140 | India | Spice | 2005 |
| *C. sakazakii* | HPB5174 | Ireland | Environment |  |
| *C. sakazakii* | 693 | France | Clinical | 1994 |
| *C. sakazakii* | 700 | France | Clinical | 1994 |
| *C. sakazakii* | 713 | France | Infant formula | 1994 |
| *C. sakazakii* | 714 | France | Infant formula | 1994 |
| *C. sakazakii* | 715 | France | Infant formula | 1994 |
| *C. sakazakii* | 2087 | France | Environmental |  |
| *C. sakazakii* | ES15 | Korea | Whole grain |  |
| *C. sakazakii* | 150 | Korea | Spice | 2005 |
| *C. sakazakii* | 2106 | Belgium | Clinical |  |
| *C. sakazakii* | 2161 | Mexico | Environmental | 2010 |
| *C. malonaticus* | 1569 | USA | Clinical | 2011 |
| *C. malonaticus* | 1846 | Czech Republic | Ingredient | 2010 |
| *C. malonaticus* | 687 | Czech Republic | Clinical | 2004 |
| *C. malonaticus* | 681 (LMG23826^T^) | USA | Clinical | 1977 |
| *C. malonaticus* | 2045 | France | Environmental |  |
| *C. malonaticus* | 2046 | France | Environmental |  |
| *C. malonaticus* | 685 | USA | Clinical | 1977 |
| *C. malonaticus* | 510 | Czech Republic | Food | 1985 |
| *C. malonaticus* | 1558 | Czech Republic | Clinical |  |
| *C. malonaticus* | CMCC45402 | China | Milk |  |
| *C. malonaticus* | 1545 | Czech Republic | Clinical |  |
| *C. malonaticus* | 507 | Czech Republic | Clinical | 1984 |
| *C. malonaticus* | 2109 | Canada | Unknown |  |
| *C. turicensis* | LMG23827^T^ | Switzerland | Clinical | 2005 |
| *C. turicensis* | 564 | USA | Clinical | 1970 |
| *C. turicensis* | 1880 | Czech Republic | Herb | 2011 |
| *C. turicensis* | 1553 | Slovakia | Unknown |  |
| *C. turicensis* | 92 | UK | Herb | 2004 |
| *C. turicensis* | 1554 | Slovakia | Unknown |  |
| *C. dublinensis* subsp. *dublinensis* | NTU 1210 (LMG 23823^T^) | Ireland | Environment | 2004 |
| *C. dublinensis* subsp. *lactaridi* | LMG23825 | Zimbabwe | Environment | 2003 |
| *C. dublinensis* subsp. *lausanensis* | LMG23824 | Switzerland | Water | 2004 |
| *C. dublinensis* | 582 | UK | Unknown |  |
| *C. dublinensis* | 583 | UK | Environment | 1956 |
| *C. dublinensis* | 1556 | USA | Clinical | 1979 |
| *C. dublinensis* | 1560 | Czech Republic | Food |  |
| *C. dublinensis* | 2030 | France |  |  |
| *C. muytjensii* | 16 | Unknown | Spice | 2005 |
| *C. muytjensii* | ATCC 51329^T^ | Unknown | Unknown |  |
| *C. muytjensii* | 530 | Denmark |  |  |
| *C. universalis* | NTU 581 (NCTC9529^T^) | UK | Water | 1956 |
| *C. condimenti* | 1330^T^ | Slovakia | Food | 2010 |

NTU= Nottingham Trent University culture collection

Table S2:Genbank accession numbers used for O-antigen loci

| **Species** | **O-type** | **Strain** | **Accession Number** |
| --- | --- | --- | --- |
| *C. sakazakii* | O1 | NCTC 11467 | EU076545 |
|  | O2 | NCTC 8155 | EU076546 |
|  | O3 | 2156 | HQ646168 |
|  | O4 | G2594 | JQ674747 |
|  | O5 | G2706 | JQ674748 |
|  | O6 | G2704 | JQ674749 |
|  | O7 | G2592 | JQ674750 |
| *C. turicensis* | O1 | z3032 | HQ646166 |
|  | O2 | G3882 | JQ354993 |
|  | O3 | E609 | JX475926 |
| *C. malonaticus* | O1 | E615 | HQ646167 |
|  | O2 | LMG 23826 | HQ646171 |
| *C. muytjensii* | O1 | E769 | HQ646170 |
|  | O2 | ATCC 51329 | JQ390552 |
| *C. dublinensis* | O1 | LMG 23823 | JQ390550 |
|  | O1 | LMG 23825 | JQ390549 |
|  | O2 | LMG 23824 | JQ390551 |
| *C. universalis* | O1 | NCTC 9529 | JQ390553 |

Table S3: Description of *C. muytjensii* O-antigen designations

| **Serotype** | **O-antigen gene cluster** | | | | | | | | | | | | | | | **Accession number** |
| --- | --- | --- | --- | --- | --- | --- | --- | --- | --- | --- | --- | --- | --- | --- | --- | --- |
| *C. muytjensii* O1 | ***galF*** | *rmlB* | *rmlD* | *rmlA* | *rmlC* | *fdtA* | *fdtC* | *fdtB* | *wzx* | *wdaN* | *wzy* | *wehJ* | *wehK* | *wehL* | ***gnd*** | HQ646170 |
| *C. muytjensii* O2 | ***galF*** | *rmlB* | *rmlD* | *rmlA* | *rmlC* | *weoP* | *weoQ* | *weoR* | *wzx* | *weoS* | *weoT* | *weoU* | *weoV* | *wzy* | ***gnd*** | JQ390552 |
| *C. muytjensii* O3 | ***galF*** | *rfbB* | *rmlA* | *fdtA* | *ORF1* | *ORF2* | *fdtB* | *ORF3* | *epsE* | *ORF4* | *ORF5* | *tuaG* | ***gnd*** |  |  |  |
| *C. muytjensii* O4 | ***galF*** | *rfbB* | *rfbD* | *rmlA* | *rfbC* | *fdtA* | *fdtB* | *ORF1* | *ORF2* | *ORF3* | *pglJ* | *tuaG* | ***gnd*** |  |  |  |

Table S4. Serotype and capsular profiles of *Cronobacter* species

| Species | Isolate | Sequence type | Clonal complex | *gnd* allele | *galF* allele | O-type | K-antigen type | Colanic acid type | Cellulose *bcs* genes | Enterobacterial common antigen type |
| --- | --- | --- | --- | --- | --- | --- | --- | --- | --- | --- |
| *C. sakazakii* | 658 | 1 |  | 1 | 2 | O1 | K1 | CA1 | + | ECA1 |
| *C. sakazakii* | 1218 | 1 |  | 1 | 2 | O1 | K1 | CA1 | + | ECA1 |
| *C. sakazakii* | 1536 | 1 |  | 1 | 2 | O1 | K1 | CA1 | + | ECA1 |
| *C. sakazakii* | 2064 | 1 |  | 1 | 2 | O1 | K1 | CA1 | + | ECA1 |
| *C. sakazakii* | 2089 | 1 |  | 1 | 2 | O1 | K1 | CA1 | + | ECA1 |
| *C. sakazakii* | 716 | 14 | 1 | 1 | 2 | O1 | K1 | CA1 | + | ECA1 |
| *C. sakazakii* | ATCC29544^T^ | 8 |  | 3 | 4 | O1 | K1 | CA1 | + | ECA1 |
| *C. sakazakii* | 5 | 8 |  | 3 | 4 | O1 | K1 | CA1 | + | ECA1 |
| *C. sakazakii* | 2048 | 8 |  | 3 | 4 | O1 | K1 | CA1 | + | ECA1 |
| *C. sakazakii* | 680 | 8 |  | 3 | 4 | O1 | K1 | CA1 | + | ECA1 |
| *C. sakazakii* | ES35 | 8 |  | 3 | 4 | O1 | K1 | CA1 | + | ECA1 |
| *C. sakazakii* | 2051 | 64 |  | 37 | 26 | O2 | K1 | CA2 | + | ECA1 |
| *C. sakazakii* | 520 | 12 |  | 24 | 22 | O3 | K2 | CA1 | + | ECA1 |
| *C. sakazakii* | 1249 | 31 |  | 29 | 26 | O2 | K2 | CA2 | + | ECA1 |
| *C. sakazakii* | 978 | 3 |  | 20 | 18 | O2 | K2 | CA2 | + | ECA1 |
| *C. sakazakii* | 984 | 3 |  | 20 | 18 | O2 | K2 | CA2 | + | ECA1 |
| *C. sakazakii* | 377 | 4 |  | 2 | 3 | O2 | K2 | CA2 | + | ECA1 |
| *C. sakazakii* | 6 | 4 |  | 2 | 3 | O2 | K2 | CA2 | + | ECA1 |
| *C. sakazakii* | 20 | 4 |  | 2 | 3 | O2 | K2 | CA2 | + | ECA1 |
| *C. sakazakii* | 553 | 4 |  | 19 | 3 | O2 | K2 | CA2 | + | ECA1 |
| *C. sakazakii* | 557 | 4 |  | 2 | 3 | O2 | K2 | CA2 | + | ECA1 |
| *C. sakazakii* | 1533 | 4 |  | 2 | 3 | O2 | K2 | CA2 | + | ECA1 |
| *C. sakazakii* | 1537 | 4 |  | 2 | 3 | O2 | K2 | CA2 | + | ECA1 |
| *C. sakazakii* | 1542 | 4 |  | 2 | 3 | O2 | K2 | CA2 | + | ECA1 |
| *C. sakazakii* | 1587 | 4 |  | 2 | 3 | O2 | K2 | CA2 | + | ECA1 |
| *C. sakazakii* | 691 | 4 |  | 2 | 3 | O2 | K2 | CA2 | + | ECA1 |
| *C. sakazakii* | 692 | 4 |  | 2 | 3 | O2 | K2 | CA2 | + | ECA1 |
| *C. sakazakii* | 694 | 4 |  | 2 | 3 | O2 | K2 | CA2 | + | ECA1 |
| *C. sakazakii* | 695 | 4 |  | 2 | 3 | O2 | K2 | CA2 | + | ECA1 |
| *C. sakazakii* | 698 | 4 |  | 2 | 3 | O2 | K2 | CA2 | + | ECA1 |
| *C. sakazakii* | 701 | 4 |  | 2 | 3 | O2 | K2 | CA2 | + | ECA1 |
| *C. sakazakii* | 702 | 4 |  | 2 | 3 | O2 | K2 | CA2 | + | ECA1 |
| *C. sakazakii* | 705 | 4 |  | 2 | 3 | O2 | K2 | CA2 | + | ECA1 |
| *C. sakazakii* | 706 | 4 |  | 2 | 3 | O2 | K2 | CA2 | + | ECA1 |
| *C. sakazakii* | 707 | 4 |  | 2 | 3 | O2 | K2 | CA2 | + | ECA1 |
| *C. sakazakii* | 709 | 4 |  | 2 | 3 | O2 | K2 | CA2 | + | ECA1 |
| *C. sakazakii* | 711 | 4 |  | 2 | 3 | O2 | K2 | CA2 | + | ECA1 |
| *C. sakazakii* | 712 | 4 |  | 2 | 3 | O2 | K2 | CA2 | + | ECA1 |
| *C. sakazakii* | 767 | 4 |  | 2 | 3 | O2 | K2 | CA2 | + | ECA1 |
| *C. sakazakii* | 1105 | 4 |  | 2 | 3 | O2 | K2 | CA2 | + | ECA1 |
| *C. sakazakii* | 721 | 4 |  | 2 | 3 | O2 | K2 | CA2 | + | ECA1 |
| *C. sakazakii* | 730 | 4 |  | 2 | 3 | O2 | K2 | CA2 | + | ECA1 |
| *C. sakazakii* | 1219 | 4 |  | 2 | 3 | O2 | K2 | CA2 | + | ECA1 |
| *C. sakazakii* | 1220 | 4 |  | 2 | 3 | O2 | K2 | CA2 | + | ECA1 |
| *C. sakazakii* | 1221 | 4 |  | 2 | 3 | O2 | K2 | CA2 | + | ECA1 |
| *C. sakazakii* | 1225 | 4 |  | 2 | 3 | O2 | K2 | CA2 | + | ECA1 |
| *C. sakazakii* | 1231 | 4 |  | 2 | 3 | O2 | K2 | CA2 | + | ECA1 |
| *C. sakazakii* | 558 | 4 |  | 2 | 3 | O2 | K2 | CA2 | + | ECA1 |
| *C. sakazakii* | SP291 | 4 |  | 2 | 3 | O2 | K2 | CA2 | + | ECA1 |
| *C. sakazakii* | G-2151 | 4 |  | 2 | 3 | O2 | K2 | CA2 | + | ECA1 |
| *C. sakazakii* | 4 | 15 | 4 | 2 | 3 | O2 | K2 | CA2 | + | ECA1 |
| *C. sakazakii* | ES713 | 218 | 4 | 2 | 3 | O2 | K2 | CA2 | + | ECA1 |
| *C. sakazakii* | 1240 | 4 |  | 14 | 13 | O4 | K2 | CA2 | + | ECA1 |
| *C. sakazakii* | 690 | 12 |  | 14 | 13 | O4 | K2 | CA2 | + | ECA1 |
| *C. sakazakii* | 696 | 12 |  | 14 | 13 | O4 | K2 | CA2 | + | ECA1 |
| *C. sakazakii* | 699 | 12 |  | 14 | 13 | O4 | K2 | CA2 | + | ECA1 |
| *C. sakazakii* | 708 | 12 |  | 14 | 13 | O4 | K2 | CA2 | + | ECA1 |
| *C. sakazakii* | 2107 | 12 |  | 14 | 13 | O4 | K2 | CA2 | + | ECA1 |
| *C. sakazakii* | 703 | 12 |  | 14 | 13 | O4 | K2 | CA2 | + | ECA1 |
| *C. sakazakii* | E764 | 12 |  | 14 | 13 | O4 | K2 | CA2 | + | ECA1 |
| *C. sakazakii* | 140 | 40 | 45 | 14 | 13 | O4 | K2 | CA2 | + | ECA1 |
| *C. sakazakii* | HPB5174 | 40 | 45 | 14 | 13 | O4 | K2 | CA2 | + | ECA1 |
| *C. sakazakii* | 693 | 13 |  | 28 | 25 | O2 | K2 | CA2 | CL- | ECA1 |
| *C. sakazakii* | 700 | 13 |  | 28 | 25 | O2 | K2 | CA2 | CL- | ECA1 |
| *C. sakazakii* | 713 | 13 |  | 28 | 25 | O2 | K2 | CA2 | CL- | ECA1 |
| *C. sakazakii* | 714 | 13 |  | 28 | 25 | O2 | K2 | CA2 | CL- | ECA1 |
| *C. sakazakii* | 715 | 13 |  | 28 | 25 | O2 | K2 | CA2 | CL- | ECA1 |
| *C. sakazakii* | 2087 | 100 |  | 13 | 1 | O1 | K2 | CA1 | CL- | ECA1 |
| *C. sakazakii* | ES15 | 125 | 100 | 13 | 1 | O1 | K2 | CA1 | CL- | ECA1 |
| *C. sakazakii* | 150 | 148 | 16 | 23 | 21 | O1 | K2 | CA1 | + | ECA1 |
| *C. sakazakii* | 2106 | 257 | 21 | 21 | 19 | O1 | K2 | CA1 | + | ECA1 |
| *C. sakazakii* | 2161 | 297 |  | 18 | 17 | O1 | K2 | CA1 | + | ECA1 |
| *C. malonaticus* | 1569 | 307 | 112 | 33 | 6 | O1 | K2 | CA2 | + | ECA2 |
| *C. malonaticus* | 1846 | 60 |  | 27 | 24 | O1 | K2 | CA2 | + | ECA2 |
| *C. malonaticus* | 687 | 60 |  | 27 | 24 | O1 | K2 | CA2 | + | ECA2 |
| *C. malonaticus* | 681 (LMG23826^T^) | 7 |  | 4 | 5 | O2 | K1 | CA1 | + | ECA2 |
| *C. malonaticus* | 510 | 7 |  | 4 | 5 | O2 | K1 | CA1 | + | ECA2 |
| *C. malonaticus* | 1558 | 7 |  | 4 | 5 | O2 | K1 | CA1 | + | ECA2 |
| *C. malonaticus* | CMCC45402 | 7 |  | 4 | 5 | O2 | K1 | CA1 | + | ECA2 |
| *C. malonaticus* | 1545 | 84 | 7 | 4 | 5 | O2 | K1 | CA1 | + | ECA2 |
| *C. malonaticus* | 2045 | 302 |  | 36 | 32 | O2 | K1 | CA1 | + | ECA2 |
| *C. malonaticus* | 2046 | 302 |  | 36 | 32 | O2 | K1 | CA1 | + | ECA2 |
| *C. malonaticus* | 685 | 129 |  | 26 | 23 | O2 | K1 | CA1 | + | ECA2 |
| *C. malonaticus* | 507 | 11 |  | 5 | 6 | O3 | K1 | CA2 | + | ECA1 |
| *C. malonaticus* | 2109 | 300 |  | 38 | 24 | O3 | K1 | CA2 | + | ECA2 |
| *C. turicensis* | LMG23827^T^ | 19 | 24 | 8 | 9 | O1 | K1 | CA2 | + | ECA2 |
| *C. turicensis* | 564 | 5 |  | 6 | 7 | O4 | K1 | CA2 | + | ECA2 |
| *C. turicensis* | 1880 | 344 |  | 34 | 30 | O3 | K1 | CA2 | + | ECA2 |
| *C. turicensis* | 1553 | 72 |  | 30 | 27 | O3 | K1 | CA2 | + | ECA2 |
| *C. turicensis* | 92 | 35 |  | 22 | 20 | O3 | K2 | CA2 | + | ECA2 |
| *C. turicensis* | 1554 | 342 |  | 31 | 28 | O1 | K2 | CA2 | + | ECA2 |
| *C. dublinensis* subsp. *dublinensis* | 1210  (LMG23823^T^) | 106 |  | 12 | 12 | O1b | K1 | CA1 | + | ECA2 |
| *C. dublinensis* subsp. *lactaridi* | LMG23825 | 79 |  | 15 | 14 | O1a | K1 | CA1 | + | ECA2 |
| *C. dublinensis* subsp. *lausanensis* | LMG23824 | 80 |  | 11 | 11 | O2 | K1 | CA2 | + | ECA2 |
| *C. dublinensis* | 1556 | 80 |  | 11 | 11 | O2 | K1 | CA2 | + | ECA2 |
| *C. dublinensis* | 582 | 36 | 80 | 11 | 11 | O2 | K1 | CA2 | + | ECA2 |
| *C. dublinensis* | 583 | 346 |  | 25 | 11 | O2 | K1 | CA2 | + | ECA2 |
| *C. dublinensis* | 1560 | 341 |  | 32 | 29 | O2 | K1 | CA2 | + | ECA2 |
| *C. dublinensis* | 2030 | 301 |  | 35 | 31 | na | K2 | CA2 | + | ECA2 |
| *C. muytjensii* | 16 | 347 |  | 10 | 10 | O3 | K1 | CA1 | + | ECA2 |
| *C. muytjensii* | ATCC51329^T^ | 81 |  | 16 | 15 | O2 | K1 | CA2 | + | ECA2 |
| *C. muytjensii* | 530 | 294 |  | 17 | 16 | O1 | K1 | CA1 | + | ECA3 |
| *C. universalis* | 581 (NCTC9529^T^) | 54 |  | 7 | 8 | O1 | K1 | CA2 | + | ECA2 |
| *C. condimenti* | 1330^T^ | 98 |  | 9 | 33 | O1 | K1 | CA2 | CL- | ECA2 |

Figure S1. Phylogenetic tree of *Cronobacter* spp. *galF* sequences (total length 501 bp)*.* DNA sequences were aligned in MEGA version 5.2 using the ClustalW algorithm. The phylogenetic trees were generated using the Maximum Likelihood method.

Figure S2. Phylogenetic tree of *Cronobacter* spp. *gnd* sequences (total length 501 bp). DNA sequences were aligned in MEGA version 5.2 using the ClustalW algorithm. The phylogenetic trees were generated using the Maximum Likelihood method.
